# Thymoma-associated multiorgan autoimmunity with periostin upregulation following PUVA-bath therapy

**DOI:** 10.1016/j.jdcr.2026.05.028

**Published:** 2026-05-20

**Authors:** Machiko Kamura, Kazunari Sugita

**Affiliations:** Division of Dermatology, Department of Internal Medicine, Faculty of Medicine, Saga University, Saga, Japan

**Keywords:** thymoma-associated multiorgan autoimmunity, thymoma, periostin, PUVA bath therapy

## Introduction

Thymoma-associated multiorgan autoimmunity (TAMA) is a rare and complex paraneoplastic autoimmune disorder that shares clinical and histopathologic features with graft-versus-host disease (GVHD).^1^ It typically arises in the setting of thymoma and manifests with involvement of the skin, gastrointestinal tract, and liver. Although the pathogenesis is not fully understood, immune dysregulation is believed to play a central role. Periostin is a matricellular protein secreted by fibroblasts in response to interleukin (IL)-4 and IL-13 and is considered a reliable biomarker of type 2 inflammation in diseases such as atopic dermatitis, asthma, and eosinophilic esophagitis.^2^ However, its role in autoimmune conditions such as TAMA has not been fully explored. We report a patient with TAMA who exhibited dynamic changes in periostin expression in both lesional skin and serum before and after PUVA-bath therapy. These findings suggest that periostin may serve as a marker of disease activity or immune polarization in TAMA.

## Case report

**Fig 1:**
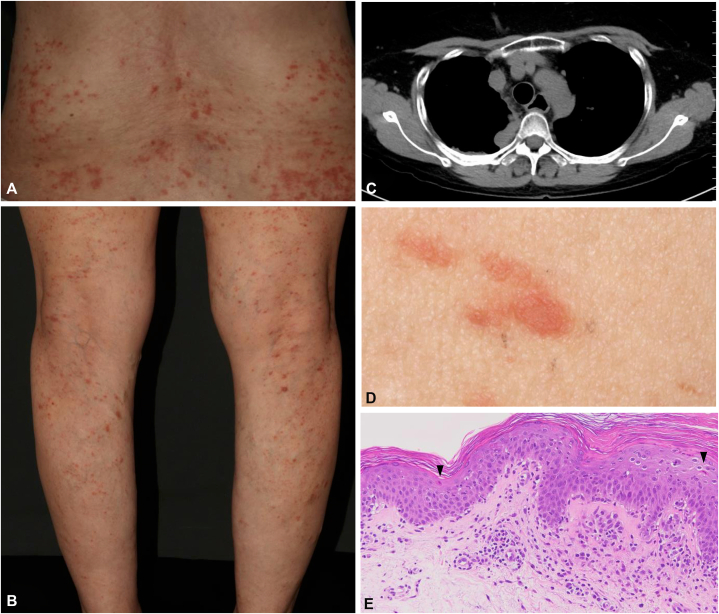
**A** and **B,** Initial presentation: multiple erythematous and papular lesions with well-demarcated borders and induration were noted on the trunk and the extremities. **C,** Chest CT scan: a nodule along the interlobar pleura of the right middle lobe demonstrated progressive enlargement, suggestive of a disseminated lesion from invasive pleuropneumonia. **D** and **E,** Histologic findings of a skin biopsy specimen from the right lateral abdomen (hematoxylin and eosin, original magnification ×200) revealed epidermal hyperplasia with irregular acanthosis, hyperkeratosis, liquefaction degeneration, and scattered necrotic keratinocytes (arrowed).

A woman in her 60s presented with widespread erythematous papules and plaques associated with severe pruritus. At the initial visit, multiple erythematous and papular lesions with well-defined borders and infiltration were present on the trunk and extremities (Fig 1, *A* and *B*). Her medical history was notable for invasive thymoma (Masaoka stage IVA, WHO type B1), diagnosed 10 y earlier. Initial treatment included 4 cycles of ADOC-P chemotherapy (cisplatin, doxorubicin, vincristine, cyclophosphamide, prednisolone), followed by surgical resection. Two years later, pleural seeding recurred, requiring further chemotherapy (Fig 1, *C*). Three years prior to this presentation, she developed myasthenia gravis and began treatment with systemic corticosteroids and immunosuppressants.

Her medications included prednisolone 17.5 mg/d, tacrolimus, alendronic acid, rebamipide, sulfamethoxazole-trimethoprim, and more recently, carbosisteine and ambroxol. A drug-induced eruption was initially suspected, and both carbocistein and ambroxol were discontinued. However, the rash progressed despite topical corticosteroid treatment. A skin biopsy from the right lateral abdomen revealed epidermal hyperplasia with irregular acanthosis, hyperkeratosis, liquefaction degeneration, and scattered necrotic keratinocytes (Fig 1, *D* and *E*). A dense lichenoid lymphocytic infiltrate with intradermal exocytosis was observed, consistent with acute GVHD-like dermatitis (Fig 1, *E*).

**Fig 2:**
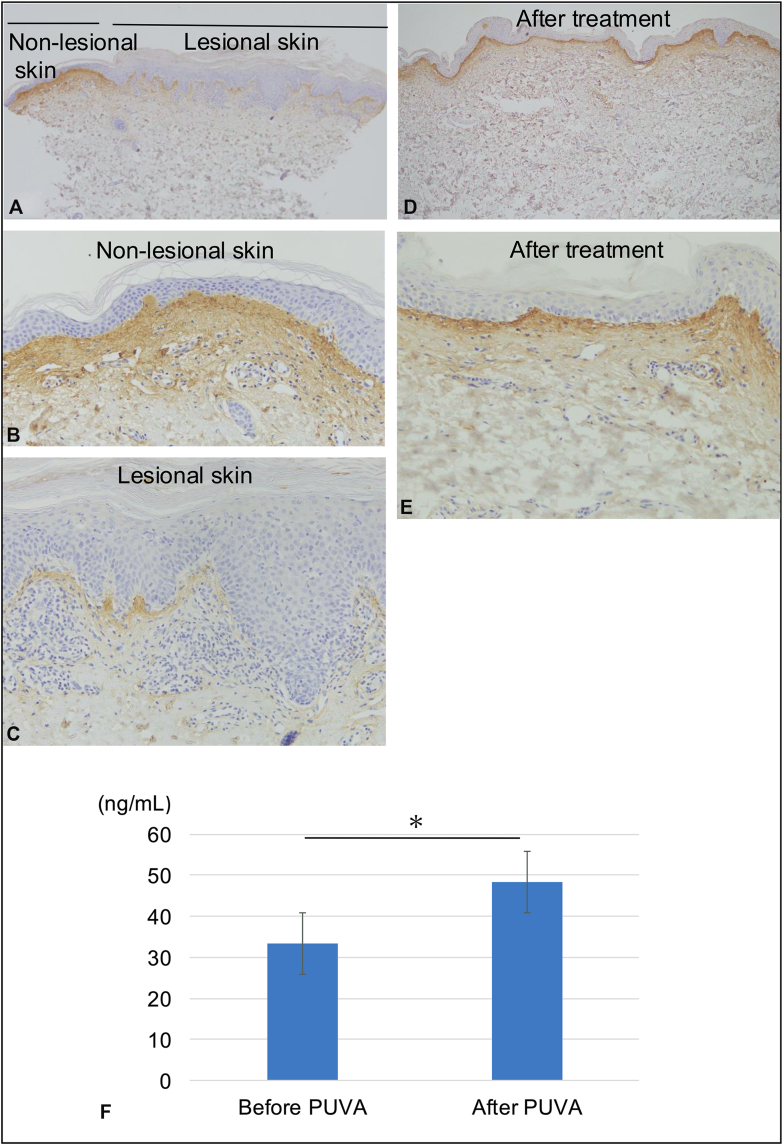
Skin biopsy prior to PUVA-bath therapy (periostin immunohistochemistry; 60 minute reaction at room temperature with heat-induced citrate antigen retrieval), original magnification ×40 **(A)**, ×100 **(B, C)**: Periostin expression was markedly reduced in the papillary dermis of lesional skin **(C)** compared to adjacent nonlesional areas **(B)**. This analysis was performed on the same tissue specimen shown in Fig 1, *D*. Skin biopsy 2 wk after PUVA-bath therapy (periostin immunohistochemistry, original magnification ×40 **[D]**, ×100 **[E])**. Periostin expression was increased in the previously affected dermis. **F,** Serum periostin levels measured before and 2 wk after PUVA-bath therapy. All measurements were performed in triplicate for each sample (technical replicates). A significant increase in periostin concentration was observed following treatment (∗*P* < .01). Mean values were 33.363 ± 0.935 ng/mL pre-treatment and 48.323 ± 2.837 ng/mL post-treatment (*P* = .001), Data represent the mean values ± SD from triplicate measurements, analyzed using a paired t-test.

Based on the patient’s history of thymoma, the progression of the rash despite discontinuation of the suspected drugs, the temporal association with thymoma progression, and the absence of granular layer thickening (typically seen in lichen planus), a diagnosis of TAMA was made. PUVA-bath therapy was initiated. A follow-up biopsy performed 2 wk later showed a marked reduction in inflammatory infiltrates. Considering its role as a marker of type 2 inflammation, periostin was examined to explore whether similar immune mechanisms might play a role in TAMA. Immunohistochemical staining revealed markedly reduced periostin expression in the papillary dermis of lesional skin prior to treatment, compared to adjacent non-lesional areas (Fig 2, *A*-*C*). Two weeks after PUVA-bath therapy, periostin expression increased in the previously affected dermis (Fig 2, *D* and *E*). Concurrently, serum periostin levels, measured 3 times in the same sample by Luminex multiplex assay as technical controls, increased from 33.4 ng/mL before treatment to 48.3 ng/mL 2 wk after treatment (Fig 2, *I*). These observations may reflect immune changes induced by treatment and prompted further interest in periostin as a potential indicator of disease status in TAMA.

## Discussion

TAMA is characterized by autoreactive T-cell responses that mimic GVHD in the absence of allogeneic transplantation.^1^ The pathogenesis involves a loss of central immune tolerance due to thymic epithelial dysfunction, leading to systemic autoimmunity.^3^ Although the cutaneous manifestations of TAMA have been well documented, there is limited information on molecular markers or immunologic indicators of disease activity.

Periostin is an extracellular matrix protein that is strongly expressed in tissues undergoing chronic type 2 inflammation.^2^ It is induced by IL-4 and IL-13 through the STAT6 signaling pathway and is commonly used as a biomarker of Th2-driven diseases such as atopic dermatitis.^2^ By contrast, previous reports have suggested Th1-dominant immune activity in TAMA, particularly in recurrent lesions.^4^ However, quantitative data on Th1/Th2 balance in TAMA remain limited. The rationale for analyzing periostin in this case was based on its known role as a marker of type 2 inflammation and tissue remodeling. Given the unclear immunological environment in TAMA, we hypothesized that periostin might reflect changes in immune polarization or therapeutic response.

Following PUVA-bath therapy, periostin expression increased in the skin, suggesting immune modulation or fibroblast reactivation. It should be noted that baseline periostin expression in normal skin is generally low. In inflammatory dermatoses such as atopic dermatitis, periostin expression is typically elevated compared with normal skin.^5^ As previously reported, periostin expression was markedly elevated in atopic dermatitis and was less pronounced in psoriasis (Supplemental Figure 1, *A*-*C*, available via Mendeley at https://data.mendeley.com/datasets/c3ckrmxdfv/1). However, previous studies have reported that periostin levels may remain elevated even after systemic treatment, possibly reflecting ongoing tissue remodeling or wound-healing processes rather than inflammatory activity alone.^6^ This may suggest that periostin dynamics do not strictly parallel inflammatory severity. In the present case, our intention is not to define a characteristic pattern of periostin expression in TAMA, but rather to describe the treatment-associated modulation observed in this individual patient. PUVA therapy has been shown to alter T-cell activity and may have facilitated a shift toward a more balanced or Th2-favoring environment, thereby promoting periostin expression and tissue remodeling.

We also observed an increase in serum periostin levels following PUVA-bath therapy in this patient with TAMA. Serum periostin levels were measured in technical triplicates at each time point to ensure assay reliability. In addition, for contextual reference, serum periostin levels were assessed using the same protocol in 1 healthy subject and in representative patients with atopic dermatitis and psoriasis (Supplemental Figure 1, *D*, available via Mendeley at https://data.mendeley.com/datasets/c3ckrmxdfv/1). Consistent with previous reports, the highest levels were observed in atopic dermatitis. These findings raise the possibility that periostin may serve not only as a biomarker of disease activity but also as an indicator of immunologic response to therapy in this rare autoimmune syndrome. However, given the limited number of cases, particularly for TAMA, the clinical significance of these findings remains to be established.

## Conflict of interest

None disclosed.
